# Multikinase and highly selective kinase inhibitors in the neoadjuvant treatment of patients with thyroid cancer

**DOI:** 10.37349/etat.2025.1002291

**Published:** 2025-02-13

**Authors:** Laura Valerio, Antonio Matrone

**Affiliations:** European University Cyprus, Cyprus; ^1^Department of Medical, Surgical and Neurological Sciences, Unit of Endocrinology, Siena University Hospital, 53100 Siena, Italy; ^2^Department of Clinical and Experimental Medicine, Unit of Endocrinology, Pisa University Hospital, 56124 Pisa, Italy

**Keywords:** Tyrosine kinase inhibitors, neoadjuvant treatment, thyroid cancer, targeted therapy, unresectable thyroid cancer

## Abstract

Multikinase inhibitors (MKIs) and highly selective tyrosine kinase inhibitors (HS-TKIs) positively impact the progression-free survival (PFS) of locally advanced and metastatic thyroid cancer cases. Moreover, disease-specific survival (DSS) and overall survival (OS) improvements were observed in some instances, suggesting a general benefit in disease control. In advanced and metastatic thyroid cancers, other conventional treatments are often ineffective when surgery cannot be performed due to the extension of the disease and/or the invasion of vital neck structures (such as the larynx, trachea, esophagus, recurrent laryngeal nerve, and carotid artery). In these cases, systemic treatments with MKIs and HS-TKIs have recently been evaluated for their potential to block tumor growth and reduce tumor size to make surgery possible or improve the control of metastatic disease. The study aimed to evaluate the performance of these systemic drugs in the neoadjuvant treatment of thyroid cancer patients, focusing on their efficacy according to the different histology.

## Introduction

The action of several drugs on tyrosine kinase receptors led to an improvement of progression-free survival (PFS) and, in some cases, disease-specific survival (DSS) and overall survival (OS) in many human cancers, including thyroid [1–3]. In thyroid cancers, these drugs are usually used in advanced metastatic disease, particularly when the disease progresses. However, in locally advanced cases characterized by the presence of large thyroid tumors with invasion of the neck structures such as the larynx, trachea, pharynx, esophagus, recurrent laryngeal nerve, carotid artery, surgery is not feasible, and the disease cannot be safely removed [4]. In such cases, surgical removal of the disease is less effective and rarely performed. External beam radiotherapy (EBRT) can slow tumor growth, but it cannot consistently shrink the tumor enough to make surgery possible. Recently, systemic treatments using multikinase inhibitors (MKIs) or highly selective tyrosine kinase inhibitors (HS-TKIs) have been explored to manage tumor growth and reduce its size. These treatments are promising for thyroid cancers that cannot be surgically removed, as they may improve outcomes by enabling tumor removal after shrinkage and controlling metastatic disease if present. The present study aims to evaluate the available literature about the use of MKIs and HS-TKIs in a neoadjuvant setting, focusing on the action of these drugs and their efficacy according to thyroid cancer’s different histology.

## Treatment of advanced thyroid cancer: what drugs we have

### Differentiated thyroid cancer

Sorafenib and lenvatinib are the only two drugs approved by the Food and Drug Administration (FDA) and the European Medicines Agency (EMA) for the first-line treatment of locally advanced or metastatic radioiodine-refractory differentiated thyroid cancer (DTC).

Sorafenib is a drug able to inhibit the RAS and BRAF/mitogen-activated protein kinase (MEK)/ERK signaling pathways; ligand-dependent REarranged during Transfection receptor (RET)/PTC receptor tyrosine kinase activation and pathways involving vascular endothelial growth factor (VEGF), platelet-derived growth factor (PDGF) and their receptors [2]. Lenvatinib is a multitargeted inhibitor of VEGF receptor (VEGFR) 1, 2, and 3, fibroblast growth factor receptor 1-4 (FGFR 1-4), PDGF receptor α (PDGFRα), RET, and v-kit Hardy Zuckerman 4 feline sarcoma viral oncogene (KIT) signaling pathways [2]. Both these drugs demonstrated a potent inhibition of VEGFR and, therefore, have been defined as antiangiogenic drugs. They have been approved according to the results of phase 3 studies in which an improvement in PFS was demonstrated against a placebo [5, 6].

Recently, cabozantinib [7], an MKI that inhibits several tyrosine kinase receptors, including hepatocyte growth factor (HGF) receptor (MET), VEGFR2, and AXL, which are involved in tumor growth, angiogenesis, and metastasis, has also been approved as second-line treatment for radioiodine-refractory advanced/metastatic DTC that has progressed after treatment with other TKIs [8].

By targeting specific molecular alterations in locally advanced or metastatic DTC that carry RET/PTC rearrangement, selpercatinib is another therapeutic option. Selpercatinib is a selective inhibitor of the RET receptor tyrosine kinase, blocking the signaling pathways that promote tumor growth and survival in RET-altered cancers [9].

Among the selective RET drugs, pralsetinib is another selective RET kinase inhibitor authorized by the FDA but not by EMA for treating advanced or metastatic RET fusion-positive DTC [8].

NTRK (neurotrophic tyrosine receptor kinase) fusions are genetic alterations characterized by the fusion of one of the NTRK genes (*NTRK1*, *NTRK2*, or *NTRK3*) with another gene, resulting in the production of a hybrid protein that can drive tumorigenesis via the RAS/RAF/MAPK pathway [10].

NTRK inhibitors can be used in both first-line and subsequent lines of therapy, depending on the specific clinical context and previous treatments [11–13]. Clinical trials have demonstrated the efficacy of these drugs across various tumor types, with durable responses in many cases. By targeting this actionable mutation, entrectinib can be used in advanced DTC patients carrying this fusion [14]. Entrectinib is designed to inhibit several kinases, including NTRK, reactive oxygen species (ROS; ROS1), and anaplastic lymphoma kinase (ALK), which are involved in cancer cell growth and survival. Entrectinib is indicated for patients with advanced or metastatic DTC harboring *NTRK* gene fusions, especially in cases refractory to standard treatments [8, 15–18].

Also, larotrectinib is a highly selective inhibitor of NTRK fusion proteins [19] involved in cell growth and differentiation and shares the same therapeutic indication as entrectinib.

In locally advanced and metastatic DTC and poorly differentiated thyroid cancer (PDTC) patients who carry the valine to the glutamic acid substitution of *BRAF* gene (BRAF^V600E^) mutation, the therapy with dabrafenib (a BRAF inhibitor) and trametinib (a MEK inhibitor) is a viable option [20–22].

Immunotherapy is an evolving treatment area for DTC, particularly for advanced cases refractory to other therapies. These agents block immune checkpoints (like PD-1, PD-L1, and CTLA-4), enhancing the immune response against cancer cells. Clinical trials are assessing the efficacy of nivolumab (anti-PD-1) and pembrolizumab (anti-PD-1) in DTC, especially in BRAF-mutant and advanced cases [23] (ClinicalTrial.gov—NCT05852223, NCT02973997, NCT03246958, NCT03914300).

Combining immunotherapy with MKIs or other targeted drugs is under investigation to enhance therapeutic effectiveness and overcome resistance.

### Anaplastic thyroid cancer

MKIs and immunotherapy have been combined for advanced metastatic anaplastic thyroid cancer (ATC) [23–29]. A few reports have demonstrated that combining MKIs and pembrolizumab can impact survival [23, 25, 26]. The use of HS-TKIs is based on the presence of specific gene mutations in the ATC. Indeed, molecular biology plays a pivotal role in the treatment options of advanced and metastatic ATC patients.

In almost all published reports, lenvatinib plus pembrolizumab has been used as adjuvant treatment for metastatic ATC after surgical treatment and/or radiotherapy and chemotherapy.

The other two drugs used in ATC patients are dabrafenib and trametinib. These drugs act sequentially by blocking the MAP kinase pathway’s RAF/MEK/ERK. Their use on BRAF^V600E^-mutated ATC patients has been promising [30–32]. Indeed, combining trametinib with dabrafenib enhances the therapeutic effect, as it addresses resistance mechanisms that may arise with BRAF inhibition alone. Moreover, larotrectinib and entrectinib can also be used in ATC if NTRK fusions are found [33, 34].

### Medullary thyroid cancer

Two MKIs were approved for treating advanced metastatic medullary thyroid cancer (MTC) cases according to the phase 3 studies: vandetanib [35] and cabozantinib [36]. In some cases, according to a phase 2 study [37], the salvage therapy with lenvatinib has been used with some success [38]. Developing drugs specifically targeting RET mutations has recently marked a new era in treating advanced MTC cases, both in patients previously treated with MKIs and in naive patients [39–41]. FDA and EMA have approved selpercatinib for treating RET mutant advanced/metastatic MTC patients who require systemic therapy. Also, pralsetinib, another potent, highly selective RET inhibitor, was previously approved by the FDA according to a phase 1/2 study [42, 43]. Still, recently, the manufacturers involved in its development have chosen to withdraw the indication for advanced/metastatic MTC; therefore, it is no longer available.

**Figure 1:**
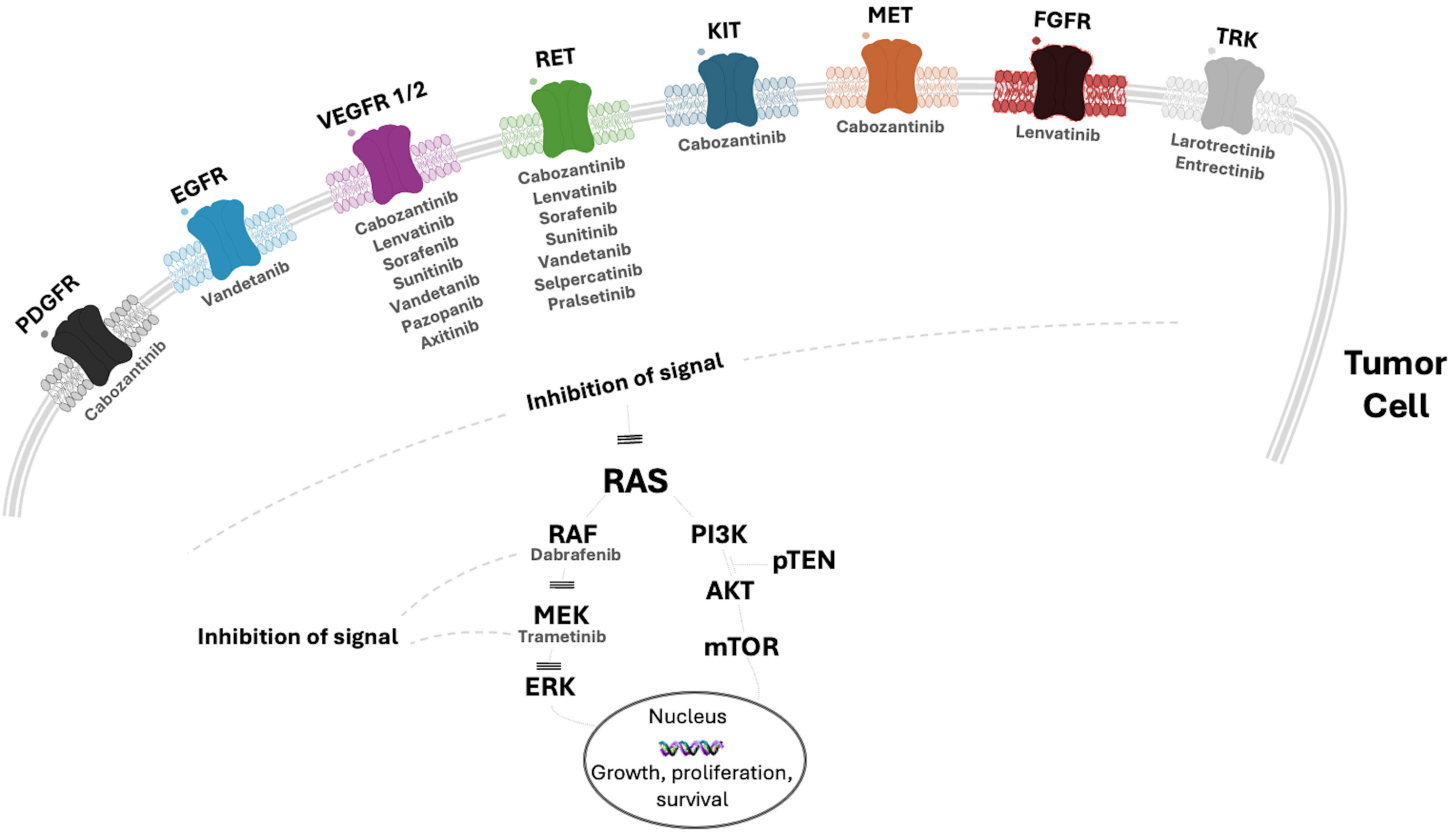
**Overview of the main tyrosine kinase receptors targeted by the most used tyrosine kinase inhibitors for the treatment of advanced thyroid cancer**. EGFR: epidermal growth factor receptor; VEGFR: vascular endothelial growth factor receptor; KIT: v-kit Hardy Zuckerman 4 feline sarcoma viral oncogene; MET: hepatocyte growth factor (HGF) receptor; RET: REarranged during Transfection receptor; FGFR: fibroblast growth factor receptor; MEK: mitogen-activated protein kinase; PDGFR: platelet-derived growth factor receptor; TRK: tropomyosin receptor kinase; pTEN: phosphatase and tensin homolog

Figure 1 and Table 1, respectively, report an overview of the tyrosine kinase receptors targeted by the available drugs and each drug’s half-maximal inhibitory concentration (IC_50_).

**Table 1:** The half-life of the main drugs used in clinical practice to treat advanced thyroid cancer and the half-maximal inhibitory concentration (IC_50_) of these drugs against the most common tyrosine kinase receptors

Drug	Half-life	RET	PDGFR	FGFR	EGFR	VEGFR	BRAF^V600E^	NTRK	Other molecular targets
Lenvatinib	28 h	35 nM	39 nM	46 nM	-	22; 4; 5.2 nM*	-	-	c-KIT, KIF5B-RET, CCDC6-RET, NcoA4-RET
Sorafenib	25–48 h	47 μM	57 μM	-	-	9; 28; 7 μM *	38 μM	-	c-KIT, FLT3
Cabozantinib	55 h	4 nM	234 nM	-	-	12; 0.035; 6 nM*	-	-	c-KIT, MET, KIF5B-RET
Selpercatinib	32 h	0.4 nM	-	-	-	0.92–67.8 nM**	-	-	-
Vandetanib	19 days	130 nM	-	-	500 nM	40; 110 nM^#^	-	-	RET-KIF5B
Pralsetinib	14.7 h	0.4 nM	-	-	-	35 nM°	-	-	KIF5B-RET, CCDC6-RET, FLT3
Entrectinib	20–40 h	-	-	-	-	-	-	0.002; 0.00057; 0.0011 nM^^^	ROS, ALK
Larotrectinib	3 h	-	-	-	-	-	-	6.5; 8.1; 10.6 nM^^^	-
Dabrafenib	8 h	-	-	-	-	-	0.5 nM	-	-
Trametinib	127 h	-	-	-	-	-	0.48 nM	-	MEK

RET: REarranged during Transfection receptor; PDGFR: platelet-derived growth factor receptor; FGFR: fibroblast growth factor receptor; EGFR: epidermal growth factor receptor; VEGFR: vascular endothelial growth factor receptor; BRAF^V600E^: valine to the glutamic acid substitution of *BRAF* gene; NTRK: neurotrophic tyrosine receptor kinase; KIT: v-kit Hardy Zuckerman 4 feline sarcoma viral oncogene; KIF5B-RET, CCDC6-RET and NcoA4-RET: RET gene fusions; FLT3: Fms-like tyrosine kinase 3; MET: hepatocyte growth factor (HGF) receptor; MEK: mitogen-activated protein kinase; ROS: reactive oxygen species; ALK: anaplastic lymphoma kinase; - : no pharmacological action against these tyrosine kinase receptors. ^*^ respectively for VEGFR1, VEGFR2 and VEGFR3; ^**^ range of inhibition on VEGFR1 and VEGFR3; ^#^ respectively for VEGFR2 and VEGFR3; ° only for VEGFR2; ^ respectively for NTRK-1, NTRK-2 and NTRK-3

## Treating thyroid cancer in a neoadjuvant setting

### Anaplastic thyroid cancer

ATC is one of the rarest (1–2%) and most aggressive forms of thyroid cancer, characterized by rapidly growing tumors with a poor prognosis (median survival—5 months) [44, 45].

The clinical presentation of ATC is characterized by symptoms like neck swelling, difficulty swallowing, and hoarseness and by fast progression and high mortality rate. If possible, the treatment of ATC is surgery following radiotherapy and/or chemotherapy [29, 46]; however, only in a few cases a complete surgical resection (R0) is possible because of the frequent invasion of critical structures of the neck (i.e., larynx, trachea, pharynx, esophagus, recurrent laryngeal nerve, and carotid artery). In some cases, despite incomplete surgery, the following radiotherapy and/or chemotherapy can improve the prognosis [24, 29, 47]. According to the severity of the disease, supportive care to manage symptoms and improve quality of life is crucial for ATC patients.

Because of the disease’s high aggressiveness, it is crucial to make the correct diagnosis [48] and set up treatment as soon as possible. Molecular biology's role in finding the potential driver mutations of ATC has become a pivotal point in treating this tumor.

Romei et al. [48] evaluated the molecular profile of 21 ATC and 21 PDTC. This analysis showed that ATC had a higher prevalence of TP53 and TERT mutations (47.6% and 42.8%, respectively), while in PDTC, TERT and BRAF mutations were the most prevalent (33.3% and 19%, respectively). Moreover, genetic heterogeneity (> 2 mutations) was more frequent in ATC (28.6%) compared with PDTC (4.7%) (*P* = 0.03). The authors concluded that ATC and PDTC may be characterized by different clinical, pathological, and genetic profiles; in particular, ATC, but not PDTC, were positive for TP53 and phosphatase and tensin homolog (PTEN) alterations, and genetic heterogeneity was more frequent in ATC than PDTC.

In a larger series, Pozdeyev et al. [49] also evaluated the genetic profiles of 583 advanced DTC and 196 ATC. The two most frequently mutated genes in ATC were *TP53* (65%) and *TERT* (65%). Moreover, 41% of *ATC* had *BRAF* gene mutations, and 27% had *RAS* gene mutations. ATC had more genetic alterations per tumor compared to other thyroid cancer histotypes, and DNA mismatch repair deficit and activity of APOBEC cytidine deaminases were identified as mechanisms associated with a high mutational burden.

Therefore, a specific drug should be started if an actionable mutation is detected. BRAF^V600E^ is the most common molecular alteration found in ATC [50] with a variable rate according to the different series. Because of this high prevalence of BRAF^V600E^ in ATC in several tertiary referral centers, the search for this mutation is performed simultaneously with histology by immunohistochemistry [29, 51]. In case of a lack of BRAF^V600E^ mutation, the American Thyroid Association (ATA) ATC guidelines [29] recommend evaluating other somatic mutations/fusions. This approach offers the potential for surgical treatment in tumors previously defined as unresectable.

#### BRAF^V600E^ mutant ATC

The BRAF^V600E^ mutation occurs in 25–45% of ATC [52–54]. In ATC BRAF^V600E^ mutant patients, only in a few cases was the association between lenvatinib and pembrolizumab used with neoadjuvant intent.

Barbaro et al. [55] reported a single case of ATC BRAF^V600E^ mutant, treated with lenvatinib (14 mg/day) and pembrolizumab (200 mg every 3 weeks) as neoadjuvant therapy, followed by a complete surgical resection of the tumor. The patient was disease-free at 1 year of follow-up [55].

Zhao et al. [56] reported a series of 57 patients with BRAF^V600E^-mutant ATC divided into three groups: 1) *neoadjuvant plus surgery*, defined as definitive surgery performed after BRAF/MEK-directed therapy; 2) *no surgery*, defined as patients who performed BRAF-directed therapy but were not treated with surgery, and 3) *upfront surgery*, defined as patients in whom surgery was performed before BRAF/MEK-directed therapy. Several BRAF inhibitors were used in this study: vemurafenib, dabrafenib, encorafenib. Several MEK inhibitors were also used: cobimetinib, trametinib, and binimetinib. The survival rate was 35.2 months in the neoadjuvant plus surgery vs. 33.2 months in the no surgery group, without significant differences. In the second group, surgery was not performed due to progression/inadequate response, poor performance status, and patient’s request. In the neoadjuvant plus surgery group the median OS was not reached; PFS was 34.2 months. OS at 12 months and 24 months was 93.6% and 80.3%, respectively, while PFS at 12 months and 24 months was 84.4% and 62.2%. One patient had a complete response. In the no surgery group, the median OS was 11.4 months, and the PFS was 5.8 months. OS at 12 months and 24 months was 38.5% and 15.4%, while PFS at 12 months and 24 months was 15.4% and 0%. Lastly, the median OS in the upfront surgery group was 48.1 months, and PFS was 14.7 months. OS at 12 months and 24 months was 74.1%, and PFS at 12 months and 24 months was 50% and 41.7% [56].

An emerging area of interest is using dabrafenib and trametinib as neoadjuvant therapy in ATC. Preliminary studies and case reports suggest that this combination can lead to significant tumor regression in patients with BRAF-mutant ATC. Some trials specifically evaluate this combination as a neoadjuvant strategy. In 2018, Cabanillas et al. [57] reported the first case of unresectable BRAF^V600E^-mutant ATC in which a neoadjuvant approach with dabrafenib, trametinib, and pembrolizumab was used with a following complete tumor surgical resection and a survival of 16 months.

Wang et al. [52] reported the use of dabrafenib and trametinib as neoadjuvant therapy in six patients with BRAF^V600E^-mutant ATC. In this report, all patients received dabrafenib plus trametinib, followed by surgical treatment with complete resection and adjuvant chemoradiation. Moreover, three patients also received pembrolizumab. In these patients, the OS was 100% at six months and 83% at one year; the locoregional disease control rate was 100%. Two patients died of metastatic disease after 8 months and 14 months, respectively, and the other four patients had no evidence of disease during follow-up.

Only scanty data regarding the association between MKIs/HS-TKIs and immunotherapy as neoadjuvant treatment in ATC patients are available. Song et al. [58] reported a case series of 18 ATC patients with (*n* = 9) or without (*n* = 9) BRAF mutation (stage IVB and IVC) treated with MKIs/HS-TKIs (dabrafenib/trametinib, lenvatinib, or anlotinib) in combination with immunotherapy (pembrolizumab, sintilimab or camrelizumab). OS, PFS, response rate (RR), and R1/R0 resection feasibility were evaluated. In the BRAF-mutated group, 8 patients were treated with dabrafenib/trametinib plus immunotherapy, and 1 patient was treated with anlotinib plus immunotherapy. In the BRAF-non-mutated group, 6 patients were treated with lenvatinib plus immunotherapy and 2 patients were treated with anlotinib plus immunotherapy. The median OS was 14 months in the whole group with one-year survival rate of 55.6%. The median OS was not reached in BRAF-mutated ATC and was longer than non-mutated ATC (*P* =0.049). Moreover, the median OS was longer in patients treated with dabrafenib/trametinib than those treated with lenvatinib or anlotinib. The morphological response was evaluated in 15 patients: 5 patients showed a complete response, 6 patients showed a partial response, 1 patient showed a stable disease, and 3 patients showed a progressive disease. The other 3 patients died before the first morphological assessment. The best objective response rate (ORR) was 61.1% and the following surgical treatment was performed in 7 patients (38.9%). Among these 7 patients, 4 experienced R0 and 3 experienced R1 resection. This paper emphasized that the combination of MKIs/HS-TKIs plus immunotherapy in neoadjuvant setting is safe and effective, particularly in BRAF-mutated ATC patients [58].

#### NTRK mutant ATC

The NTRK fusions are rarely found in solid tumors, including thyroid cancers; however, they may represent a molecular target for therapy. In three clinical trials, larotrectinib was evaluated in tropomyosin receptor kinase (TRK) fusion-positive thyroid tumors [11–13]. A combined analysis of two of these trials (NCT02122913 and NCT02576431) included 28 patients with locally advanced or metastatic thyroid cancers [19 patients with papillary thyroid cancer (PTC), 7 patients with ATC, and 2 patients with follicular thyroid cancer (FTC)]. An objective response was observed in three patients with ATC (two showed a partial response, and one had a stable disease) [33].

Only five patients with thyroid carcinoma were included in an integrated analysis of three ongoing early-phase trials [59] in metastatic or locally advanced solid tumors harboring oncogenic *NTRK1*, *NTRK2*, and *NTRK3* gene fusions treated with entrectinib. However, the report did not include the tumor’s histological characteristics. Of note, larotrectinib and entrectinib showed a good safety profile, and adverse events were easily manageable, mainly in grades 1–2, according to CTCAE (Common Terminology Criteria for Adverse Events) [60].

Damásio et al. [34] reported a case of an unresectable ATC initially treated with lenvatinib with a fast shrinkage of the tumor and a following disease progression after 12 weeks. Because of the *ETV6-NTRK3* mutation, entrectinib was started with a morphological response. After 1 year of treatment with entrectinib, the patient was treated with total thyroidectomy and central compartment lymph node dissection. Subsequent radiotherapy and chemotherapy were performed, followed again by entrectinib therapy with no evidence of disease 3 months after combined treatment.

#### ATC without actionable mutation

Finding actionable mutations in ATC patients is significant, even for neoadjuvant treatment, because targeted therapy can significantly improve their prognosis. However, ATC cases without actionable mutations still require treatment. In wild-type ATC, MKIs therapy has been explored. Lenvatinib has been used as a neoadjuvant therapy, achieving only a 2.2-month survival increase compared to palliative chemotherapy [61, 62].

Another case report of unresectable wild-type ATC with PD-L1 expression > 90% was treated with lenvatinib and pembrolizumab for 1 month, allowed tumor resection, and achieved disease stability for at least 11 months [27].

Some clinical studies used camrelizumab (SHR-1210), a humanized, high-affinity, selective IgG4-κ anti-PD-1 monoclonal antibody, demonstrating promising efficacy and acceptable safety in other solid tumors [63].

Yang et al. [64] reported a clinical case of a wild-type ATC patient treated with neoadjuvant famitinib and camrelizumab, achieving complete resection, locoregional control, and survival of 24 months after diagnosis.

Further investigation into new therapeutic targets, which could be a promising neoadjuvant treatment for ATC, is necessary to improve the impact on these patients’ survival and quality of life.

### Differentiated thyroid cancer

Unlike ATC, which is a rare tumor, DTC is the most frequently diagnosed thyroid cancer. According to histologic features, it can be divided into two main subtypes: PTC and FTC. PTC is the most common, accounting for about 80% of thyroid cancers. It typically grows slowly and is often diagnosed at an early stage with a good prognosis, so much so that active surveillance strategies are currently the standard of care in selected low-risk cases [65–67].

FTC accounts for 10–15% of thyroid cancers and is more aggressive than PTC because it can spread through blood vessels to distant sites like the lungs and bones. It is often found in older adults. Treatment of DTC usually starts with surgery according to the clinical presentation of the tumor: hemi or total thyroidectomy with or without prophylactic or therapeutic lymph node dissection [68]. Radioactive iodine therapy after surgery is now allowed for adjuvant or therapeutic purposes [69] and is usually performed in cases at intermediate-high or high risk of recurrence [68]. These cancers are usually treatable with surgery, achieving complete removal (R0) in most cases. MKIs or HS-TKIs are used only for patients with advanced or metastatic disease when standard treatments are no longer effective. In these cases, several drugs are tested [2], but only sorafenib and lenvatinib are approved as first-line treatments. Little evidence is available regarding the MKIs and HS-TKIs used as neoadjuvant treatments for DTC to improve surgical outcomes in those unresectable cases. Lenvatinib, as a neoadjuvant treatment, may be considered in selected patients with aggressive or advanced DTC or PDTC before surgery. In these cases, this drug can help to reduce tumor burden and improve subsequent surgical treatment [70, 71]. Infiltration of vital neck structures like the trachea or esophagus can increase the risk of fistulas or organ perforation but is not a strict contraindication for treatment. While EBRT isn’t strongly linked to these complications, it may still contribute to the risk of perforation. The anti-angiogenic effect of lenvatinib can lead to fistulas by necrosis of the tumoral lesions [72].

A Latin American study reported using lenvatinib or sorafenib as a neoadjuvant treatment in DTC and PDTC. Patients received sorafenib (*n* = 6) or lenvatinib (*n* = 12) with a median reduction in the diameter of the primary tumor of 25% after a median of 6 months of treatment. Surgical treatment was performed in 10 patients (55%) of whom 6 cases achieved R0/R1 resection status [73].

In 2017, Tsuboi et al. [74] reported the use of lenvatinib as a neoadjuvant therapy in a 73-year-old case of advanced DTC with multiple lymph node metastases invading the right internal jugular vein, the esophagus, and trachea. Due to the complex surgical nature of the lymphadenopathy, lenvatinib at 14 mg daily was administered for 22 weeks, resulting in an 84.3% reduction in one lymph node and a 56% reduction in the other, enabling resection while preserving the esophagus. After 11 months, radioiodine treatment was performed, and no distant metastases were observed. Another case report described a DTC patient with a tumor invasion of the trachea, making it initially unresectable. Lenvatinib was administered as neoadjuvant treatment at 24 mg daily for 14 months, resulting in tumor shrinkage, which allowed for complete surgical resection of the tumor and following radioiodine treatment [75]. In 2020, Iwasaki et al. [76] reported a case of DTC localized to the mediastinum with pulmonary metastases. The mediastinal mass obstructed the brachiocephalic trunk and the superior vena cava. This patient was treated with lenvatinib as a neoadjuvant treatment at 14 mg daily for 16 weeks with relevant tumor shrinkage and subsequent total thyroidectomy and resection of the mediastinal mass. Three months after surgery, the metastatic lesions disappeared, and the mediastinal mass was completely resected. Sorafenib was used as a neoadjuvant treatment, too. Danilovic et al. [77] reported a case of 20-year-old DTC patients with an unresectable large cervical mass with severe tracheal stenosis and suspicious lung and lymph node metastases. In this patient, neoadjuvant treatment with sorafenib at 400 mg daily was started. After 13 months of treatment, there was a relevant tumor shrinkage, which allowed for near-total thyroidectomy and lymphadenectomy without achieving R0, followed by radiotherapy and radioiodine treatment that showed uptake in the cervical area and lung metastases. A total body CT scan performed 16 months after radioiodine therapy showed a persistent but stable disease in the thyroid bed, neck lymph nodes, and lung metastases. The patient performed a second treatment with radioiodine and a post-treatment whole-body scan confirming persistent radioiodine avid lesions. At the last available evaluation, 52 months after the initial diagnosis, the patient had stable metastatic disease but without relevant symptoms.

In 2019, Nava et al. [78] reported a case of unresectable DTC (7.8 cm in the largest dimension) invading the trachea and esophagus. Sorafenib was administered for 6 months, resulting in a 70% reduction in tumor size and detachment from adjacent structures. Total thyroidectomy and radioiodine treatment were performed, and after one year of follow-up, the patient is asymptomatic with a status of disease defined as an incomplete biochemical response. Although neoadjuvant therapy is not standard for most DTC cases, several clinical trials are exploring its safety and efficacy (Table 2).

**Table 2:** MKIs, HS-TKIs, and immunotherapy used as a single agent or in combination for the neoadjuvant treatment of patients with thyroid cancer inside the clinical trials that are still ongoing and/or actively recruiting participants

Drug	Tumor	Study type	Recruitment status	ClinicalTrial.gov ID
Larotrectinib	Solid tumor with documented *NTRK* gene fusion rearrangement	Phase 2	Active, not recruiting	NCT02576431
Larotrectinib	Solid tumor with documented *NTRK* gene fusion rearrangement	Phase 2	Active, not recruiting	NCT02637687
Lenvatinib	DTC, PDTC	Phase 2	Recruiting	NCT04321954
Selpercatinib	DTC, PDTC, ATC, MTC	Phase 2	Active, not recruiting	NCT04759911
Anlotinib	DTC, PDTC, MTC	Phase 2	Unknown status	NCT04309136
Camrelizumab + Apatinib	DTC, PDTC, MTC	Phase 2	Unknown status	NCT04612894
Pembrolizumab	DTC, PDTC	Phase 2	Not yet recruiting	NCT05852223
Nivolumab + Ipilimumab	DTC, ATC, MTC	Phase 2	Active, not recruiting	NCT03246958
Cabozantinib + Nivolumab + Ipilimumab	DTC, PDTC	Phase 2	Active, not recruiting	NCT03914300

MKIs: multikinase inhibitors; HS-TKIs: highly selective tyrosine kinase inhibitors; NTRK: neurotrophic tyrosine receptor kinase; DTC: differentiated thyroid cancer; PDTC: poorly differentiated thyroid cancer; ATC: anaplastic thyroid cancer; MTC: medullary thyroid cancer

Regarding lenvatinib, an ongoing clinical trial is recruiting DTC patients with invasive extrathyroidal cancer (ClinicalTrial.gov—NCT04321954). This multicenter, phase 2, open-label study examines the effect of neoadjuvant lenvatinib given to patients with extrathyroidal DTC before thyroidectomy. Lenvatinib is administered orally daily at a predetermined dose for 2, 4, or 6 cycles (1 cycle = 28 days), dependent on response. Total thyroidectomy or near-total thyroidectomy is the objective to reach after lenvatinib treatment. The main aim of this study is to evaluate the overall R0/R1 resection rate, as defined by the proportion of patients who undergo successful thyroidectomy with clear (R0) or microscopically positive surgical margins (R1). The secondary objectives are evaluating change in surgical complexity and morbidity score, the RR before surgery based on Response Evaluation Criteria in Solid Tumors version 1.1 (RECIST), the adverse events, and the conversion rate of the tumor from unresectable to resectable.

Another ongoing clinical trial is recruiting RET-mutant DTC patients with locally advanced primary tumors to use selpercatinib as a neoadjuvant treatment before surgery (ClinicalTrial.gov—NCT04759911). This phase 2 trial mainly aims to evaluate the ORR after 7 months of treatment and the rate of patients who can be successfully treated with thyroidectomy with clear (R0) or microscopically positive (R1) surgical margin. The secondary endpoints are to evaluate PFS, locoregional PFS, surgical morbidity/complexity score, OS, the incidence of adverse events, and quality of life. Patients receive selpercatinib orally twice daily on days 1–28 (1 cycle). Without disease progression or unacceptable toxicity, treatment is performed for 7 cycles. Then, patients perform surgery. After completing the study treatment, patients will be followed up to evaluate potential disease progression status every 3–4 months for the first 2 years (ClinicalTrial.gov—NCT04759911).

A phase 2 trial has been performed with anlotinib (VEGFR2 and VEGFR3 inhibitor) as neoadjuvant treatment in locally advanced DTC patients. The primary endpoint was to evaluate the ORR, while the secondary outcomes were the R0/R1 resection rate, disease control rate, OS, and incidence of adverse events. A total of 13 patients were enrolled and received anlotinib treatment (3.5 cycles) with an ORR of 76.9%, a median time to response of 61.5 days, and most patients achieved R0/R1 resection [79].

Another phase 2 study evaluated the combination therapy of surufatinib (FGFR1 inhibitor) and toripalimab (anti-PD-1 antibody) in locally advanced DTC. The surufatinib dose was 300 mg/daily once daily for a 28-day cycle. After treatment, the patients received surgical treatment if the tumor is considered resectable by clinical examination. Patients with a high risk of postoperative recurrence received radioiodine treatment. After radioiodine treatment, maintenance treatment with surufatinib was determined according to the recurrence risk stratification. The main aim of this study was to evaluate the ORR. The secondary endpoints were to evaluate R0/R1 resection rate, disease control rate, PFS and incidence of adverse events. Ten patients were enrolled in the study and received at least 4 treatment cycles. The ORR was 60%, and 9 patients showed R0/R1 resections after neoadjuvant treatment [80].

Another study is ongoing to determine the efficacy and safety of the anti-PD-1 antibody camrelizumab combined with apatinib (VEGFR2 inhibitor) for neoadjuvant therapy in locally advanced thyroid cancer. Patients received apatinib 250 mg/daily and camrelizumab 200 mg/daily, intravenous once every 2 weeks as neoadjuvant treatment for at least two cycles (1 cycle = 28 days). The primary objective of this phase 2 trial is to evaluate ORR after 24 weeks of treatment, and the secondary endpoints are to evaluate the rate of R0/R1 resection and to assess disease control rate after 6 weeks, OS up to 3 years and the incidence of adverse events (Clinicaltrial.gov—NCT04612894).

### Medullary thyroid cancer

MTC originates from parafollicular, or C cells derived from the neural crest. The peculiarity of MTC is that can be inherited in about 25% of the cases [81, 82] in an autosomal dominant way. Although it could carry several types of mutations [83–85], like DTC, in almost all familial cases of multiple endocrine neoplasia type 2A (MEN 2A) and 2B (MEN 2B) and about half of sporadic cases [85–87] mutations in *RET* gene are detected. This prevalence in sporadic cases can increase to 80% if advanced metastatic cases are considered [88]. The high prevalence of *RET* gene mutation in MTC makes this gene an ideal diagnostic and therapeutic target for MTC treatment [81, 89, 90]. Total thyroidectomy, central compartment lymph node dissection (prophylactic or therapeutic), and oriented lateral-cervical lymph node compartment dissection are the gold standard of the initial treatment of MTC [91]. The impossibility of surgical resection is an uncommon event in managing MTC; however, it can happen in some locally advanced cases. Because of the high prevalence of *RET* gene mutation in advanced MTC, the low prevalence of grades 3 and 4 adverse events [39, 41] according to CTCAE [60], and the good preservation of the quality of life, highly selective RET inhibitor drugs have been more frequently tested in a neoadjuvant setting.

In 2021, the first case of neoadjuvant salvage therapy for a locally advanced, inoperable MTC patient with selpercatinib was reported [92]. The use of selpercatinib in a neoadjuvant setting in 4 MTC cases was subsequently reported by the same group [93]. In all cases, neoadjuvant treatment was followed by surgery, and patients were followed up for a median of 2 years after selpercatinib initiation. Locoregional disease control with a reduction in surgical morbidity was obtained. Based on these results, a clinical trial was built to evaluate the efficacy of neoadjuvant selpercatinib treatment in RET mutant MTC cases (ClincalTrial.gov—NCT04759911). Very recently, a Latin American real-life experience reported a 25% tumor reduction and an R2 resection after surgery in a single patient treated with selpercatinib as first-line therapy. However, complete resection was hindered by tumor extension [73]. Differently from DTC, in whom the tracheal and/or esophageal invasion is more frequent and the risk of fistula is high, in a patient with MTC, the tumor lysis syndrome has been reported as an adverse event of neoadjuvant treatment with selpercatinib [94].

In the absence of RET mutation, therapy with MKIs has been used in a neoadjuvant setting. In the Latin American experience, Pitoia et al. [73] treated 27 patients with thyroid cancer in a neoadjuvant setting. Of these, 6 cases had MTC: 5 treated with vandetanib and 1 with selpercatinib. In this study, none had germline RET mutations, although two were positive for somatic Met918Thr RET mutation. The authors reported a median tumor diameter reduction of 24.5% after 9.5 months of treatment in the 5 patients treated with vandetanib, with a median follow-up of 50 months. The best overall response included one partial response and three cases of stable disease. However, despite this shrinkage, only one patient achieved a complete (R0/R1) resection. Grasic Kuhar et al. [95] reported their experience with MKIs used as neoadjuvant therapies in patients with advanced unresectable MTC. They treated 8 patients, 7 with sunitinib and 1 with vandetanib. The median duration of MKI treatment was 7.8 months, and the median OS of these patients was 20 months. In 4 (50%) patients, the neoadjuvant therapies led to a partial response, while 2 (25%) patients had stable disease, and the other 2 (25%) showed progressive disease in distant metastases but not in the neck. Overall, in 5/8 (62.5%) cases, the tumor became treatable by surgery after MKI treatment, but surgery was performed only in 2 cases. Sunitinib was also used for treating an unresectable MTC [96] with an initial misleading diagnosis of ATC, who failed to respond to two lines of chemotherapy plus radiotherapy. After 19 months from the beginning of sunitinib, the tumor became resectable, sunitinib was stopped 6 weeks before, and surgical treatment was performed. Histological examination revealed the tumor to be MTC rather than ATC, with no RET mutations but a homozygous Leu769Leu (2307T > G) polymorphism detected. Because of its greater potency of inhibition on VEGFR, lenvatinib was used as a single agent in treating an advanced unresectable MTC suspected of tracheal and esophageal infiltration [97]. The tumor measured 8.3 cm, and several ipsilateral left lateral cervical metastatic lymph nodes, the larger of 6.9 cm, were detected. Calcitonin levels were 32,926 pg/mL. Then, lenvatinib was initiated at a dosage of 10 mg twice daily. After about 22 weeks of therapy, a left lobectomy with ipsilateral laryngeal nerve and lateral cervical lymph node removal was performed. Notably, during surgical inspection, the esophagus, trachea, and great vessels were free of gross tumor invasion. The patient showed a significant drop in the calcitonin values and was followed up over time without any other systemic treatment.

## Neoadjuvant treatments in thyroid cancers: what we need to know

Neoadjuvant treatment is a standard of care for several types of cancers, including breast, rectal, and pancreatic [98–100]. Thyroid cancer is often treated with surgery because most cases allow for complete removal of the thyroid and neck lymph nodes. However, surgery is not feasible in some cases, especially with ATC, due to extensive tumor infiltration. According to the data of the literature and our experience as a tertiary referral center for the management of thyroid cancer, the patients are considered inoperable if they have extensive tracheal, laryngeal, esophageal infiltration, prevertebral fascia or vertebral infiltration, carotid artery infiltration or circumferential encasement and mediastinal vessels infiltration, particularly when multiple areas are affected. In such cases, surgery carries high risks, and there is a low chance of complete tumor removal (R0 resection). Extensive neck structure infiltration is the main factor limiting the R0 resection, followed by risks from comorbidities or advanced age, even with skilled surgeons. When surgery isn’t initially possible, neoadjuvant treatment may be considered to control the disease and enable future surgery. However, unlike other cancers (i.e., breast), neck infiltration risks like tracheal or esophageal fistulas and blood vessel rupture must be carefully assessed. A thorough evaluation, including clinical exams, imaging, endoscopy, and blood tests, is essential, along with multidisciplinary consultation before starting neoadjuvant therapy. The use of MKIs or HS-TKIs as neoadjuvant therapy is limited by drug availability and reimbursement in different countries. Additionally, selecting the right drug depends on identifying the tumor’s driver mutation. For this reason, in our experience, we usually perform a TRU-CUT biopsy on all thyroid tumors deemed inoperable to quickly obtain data about their histology and molecular signature [101]. The molecular data can be obtained by the next generation system (NGS), if available, or by a single laboratory standardized method able to evaluate the actionable mutation/fusions using DNA/RNA. Ideally, if the tumor has an actionable mutation, the drug of choice should be HS-TKI against that mutation. The lower antiangiogenic activity, with a potential lower chance of having fistula or blood vessel rupture and CTCAE grades 3 and 4 adverse events compared to MKIs, and the relatively similar efficacy make HS-TKIs the drugs of choice. Regarding the starting dose, if no contraindications are present, the HS-TKI should be started at the maximum suggested dose. Conversely, if the molecular data of the tumor is unavailable or the tumor has no actionable mutation detected, the MKIs become the drug of choice. Different from HS-TKIs, the initial dose of the MKIs should be tailored according to the local invasion of cancer, mainly if the trachea/esophagus or blood vessels are involved. Therefore, the maximum suggested dose is feasible if the local invasion is minimal; conversely, if the local invasion is massive, to avoid the occurrence of fistula/blood vessel rupture, a lower starting dose is suggested. The main limitation of using MKIs or HS-TKIs in neoadjuvant therapy is the risk of adverse events (Table 3). However, managing these side effects is similar to treating advanced thyroid cancer patients (Table 4) but requires extra care for specific adverse events related to the thyroid gland in situ [94]. Also, acquired resistance to these drugs, mainly studied in patients treated with HS-TKI against *RET* gene [102, 103], can limit their efficacy in treating these patients. However, the acquired resistance usually appears in the long-term follow-up of these patients. Conversely, when considering the neoadjuvant setting, the clinical response usually occurs within the first 6–12 months of therapy, making the acquired resistance a minor problem.

**Table 3:** Main adverse events classified according to the Common Terminology Criteria Adverse Events associated with MKIs and HS-TKIs treatment

Drug	Hypertension		Diarrhea		Skin rash		Anorexia		Nausea		Weight loss		Fatigue		QTc prolongation	
	**Any grade**	**G3/4**	**Any gade**	**G3/4**	**Any grade**	**G3/4**	**Any grade**	**G3/4**	**Any grade**	**G3/4**	**Any grade**	**G3/4**	**Any grade**	**G3/4**	**Any grade**	**G3/4**
Lenvatinib [5]	+++	++	+++	+	+	+	+++	+	++	+	++	+	+++	+	+	+
Sorafenib [6]	++	+	+++	+	+++	+	++	+	+	NE	++	+	++	+	NE	NE
Cabozantinib [36]	++	+	+++	+	+	+	++	+	++	+	++	+	++	+	NE	NE
Selpercatinib [41]	++	+	+++	+	+	+	+	+	+	+	+	+	+	+	NE	NE
Vandetanib [35]	++	+	+++	+	++	+	+	+	++	NE	+	NE	+	+	+	+
Pralsetinib [82]	+	+	+	+	NE	NE	NE	NE	NE	NE	NE	NE	+	+	NE	NE
Entrectinib [58]	NE	NE	++	+	+	NE	NE	NE	+	NE	NE	NE	++	+	NE	NE
Larotrectinib [13]	NE	NE	++	+	NE	NE	NE	NE	++	+	NE	NE	++	+	NE	NE
Dabrafenib + Trametinib [22]	+	+	+	NE	+	NE	+	NE	+++	+	NE	NE	+++	+	NE	NE

MKIs: multikinase inhibitors; HS-TKIs: highly selective tyrosine kinase inhibitors; QTc: QT interval corrected for heart rate; NE: no evidence; +: < 25%; ++: 25–50%; +++: 50–75%; ++++: > 75%

**Table 4:** Management of the main AEs experienced by the patients during MKIs and HS-TKIs treatment

AE	Management of AEs
Hypertension	1. ACEI, ARBs, diuretics, beta-blockers, alpha-blockers, nitrate derivates, calcium channel blockers (low interaction potential) 2. Nifedipine (use cautiously)
Diarrhea	Grade 1: oral hydration and electrolyte replacement; initiate anti-diarrheal medication (loperamide; opioids: diphenoxylate/atropine, tincture of opium); BRAT diet Grade 2: intravenous (IV) fluids if the patient is unable to tolerate oral fluids; initiate/continue anti-diarrheal as mentioned above; BRAT diet; anticholinergic agents (hyoscyamine, atropine) Persistent grades 2, 3, 4: patient hospitalization (intensive care for grade 4); provide IV fluids and use anti-diarrheal agents and anticholinergics as mentioned above; consider octreotide
Skin rash	Skin protection; urea lotion
Weight loss, anorexia, nausea	Grade 1–2: generally, do not warrant interruption of drug unless intolerable AE despite optimal management Grade 3 or intolerable adverse reactions: require interruption of the drug until resolution or improvement of AE and restart the drug at a reduced dose Grade 4: discontinue treatment in case of life-threatening reactions Intervention: 1. Nutritional supplements 2. Appetite stimulation drugs: megestrol acetate, medroxyprogesterone acetate, dexamethasone, cannabinoids 3. Antinausea drugs: metoclopramide 4. Nutrionist counseling
Fatigue	1. Screening and earlier symptoms management 2. Rate the patient level of fatigue on a scale of 0 to 10 (i.e., visual analogue scale, FACT-F) 3. Encouraged patient to maintain an active lifestyle 4. Agopunture 5. Taking MKI in the evening (rather than during the day) can minimize daytime fatigue 6. Exclude the comorbidities (anemia, hypothyroidism, hypogonadism, etc.) or electrolyte abnormalities In case of severe fatigue: 1. Psychosocial intervention and exercise 2. Management of sleep disturbances 3. Pharmacological intervention (central nervous system stimulants, antidepressant)
QTc prolongation	Grade 1 (450–480 ms): no drug interruption but careful follow-up Grade 2 (481–500 ms) and grade ≥ 3 (> 501 ms or > 60 ms compared to baseline): discontinue treatment

MKIs: multikinase inhibitors; HS-TKIs: highly selective tyrosine kinase inhibitors; ACEI: angiotensin-converting enzyme inhibitors; ARBs: angiotensin receptor blockers; BRAT diet: banana, rice, applesauce, toast; AE: adverse event; FACT-F: Functional Assessment of Cancer Therapy-Fatigue; QTc: QT interval corrected for heart rate

## Limitations of current studies and unmet needs

Neoadjuvant treatment in thyroid cancer patients has been recently reconsidered due to the development of several drugs, MKIs, and HS-TKIs in the last few years. The main limitation of using MKIs or HS-TKIs in neoadjuvant therapy is the lack of data, as most thyroid cancers are treatable with surgery. Neoadjuvant treatment is primarily used for aggressive thyroid cancer cases, mainly ATC, often managed in specialized centers with expert care and access to these drugs. This limits the general applicability of the results. Consistent data about combination therapies, particularly the association between MKIs or HS-TKIs and immunotherapy, are still lacking. Moreover, different equipes with different surgeons and expertise have judged the patients’ inoperability without a complete agreement about the criteria of inoperability. The lack of a complete molecular signature of the tumor, including the driver and other mutations and epigenetics, does not allow us to go into the details of the clinical responses. Lastly, most of the cases lacked long-term follow-up data. To overcome these limitations, several clinical trials exploring the role of neoadjuvant treatment in several types of thyroid cancers have been built and are ongoing.

## Conclusions

The efficacy of MKIs and HS-TKIs has been proved in several clinical trials and real-life experiences in managing advanced thyroid cancers. In unresectable cases, their action in a neoadjuvant setting has been reported in single or small case series cases. Although tumor shrinkage was obtained in most cases, showing the efficacy of these drugs in controlling tumor progression, conflicting results have been reported when the goal was the complete surgical removal of the tumor (R0). However, using these drugs in a neoadjuvant setting could be helpful in the clinical outcome of patients with unresectable thyroid cancers, paying attention when the tumor invades the neck structures (i.e., larynx, esophagus, trachea). The use of HS-TKI, if actionable mutations are detected, having lower anti-angiogenic activity and fewer adverse events, should be the principal option. Ongoing clinical trials and additional case studies will play a key role in refining treatment strategies and increasing the potential for curative surgery in patients with advanced unresectable thyroid carcinoma.

## Abbreviations

ATC: anaplastic thyroid cancer
BRAF^V600E^: valine to the glutamic acid substitution of *BRAF* gene
DTC: differentiated thyroid cancer
EMA: European Medicines Agency
FDA: Food and Drug Administration
FGFR 1-4: fibroblast growth factor receptor 1-4
FTC: follicular thyroid cancer
HS-TKIs: highly selective tyrosine kinase inhibitors
KIT: v-kit Hardy Zuckerman 4 feline sarcoma viral oncogene
MEK: mitogen-activated protein kinase
MET: hepatocyte growth factor receptor
MKIs: multikinase inhibitors
MTC: medullary thyroid cancer
NTRK: neurotrophic tyrosine receptor kinase
ORR: objective response rate
OS: overall survival
PDGFR: platelet-derived growth factor receptor
PDTC: poorly differentiated thyroid cancer
PFS: progression-free survival
PTC: papillary thyroid cancer
RET: REarranged during Transfection receptor
ROS: reactive oxygen species
VEGFR: vascular endothelial growth factor receptor

## Declarations

### Copyright

© The Author(s) 2025.
